# Distribution of HPV Genotypes in Patients with a Diagnosis of Anal Cancer in an Italian Region

**DOI:** 10.3390/ijerph17124516

**Published:** 2020-06-23

**Authors:** Narcisa Muresu, Giovanni Sotgiu, Laura Saderi, Illari Sechi, Antonio Cossu, Vincenzo Marras, Marta Meloni, Marianna Martinelli, Clementina Cocuzza, Francesco Tanda, Andrea Piana

**Affiliations:** 1 Department of Medical, Surgical and Experimental Sciences, University of Sassari, 07100 Sassari, Italy; narcisamuresu@outlook.com (N.M.); lsaderi@uniss.it (L.S.); 2 Department of Biomedical Science, University of Sassari, 07100 Sassari, Italy; illarisechi@yahoo.it (I.S.); piana@uniss.it (A.P.); 3 Department of Medical, Surgical and Experimental Sciences, Surgical Pathology, University of Sassari, 07100 Sassari, Italy; cossu@uniss.it (A.C.); marrasv@gmail.com (V.M.); martameloni1981@libero.it (M.M.); tandaf@uniss.it (F.T.); 4 Department of Medicine and Surgery, University of Milano-Bicocca, 20900 Monza, Italy; marianna.martinelli@unimib.it (M.M.); clementina.cocuzza@unimib.it (C.C.)

**Keywords:** HPV, anal cancer, epidemiology, p-16, E6-gene

## Abstract

*Objectives:* Anal cancer is a rare disease. However, its incidence is increasing in some population groups. Infection caused by Human Papillomavirus (HPV) is strongly associated with the risk of anal cancer, whose variability depends on samples, histology, and HPV detection methods. The aim of the study was to assess prevalence and distribution of HPV genotypes in patients diagnosed with anal carcinoma. *Methods*: An observational, retrospective study was carried out in a tertiary care hospital in North Sardinia, Italy. Specimens of anal cancer diagnosed from 2002–2018 were selected. Demographic, epidemiological, and clinical variables were collected to assess their relationship with the occurrence of anal cancer. *Results*: The overall HPV positivity was 70.0% (21/30), with HPV-16 being the predominant genotype (~85%). The highest prevalence of anal cancer was in patients aged ≥55 years. HPV positivity was higher in women (*p*-value > 0.05) and in moderately differentiated samples (G2) (*p*-value < 0.05). p16^INK4a^ and E6-transcript positivity were found in 57% and 24% of the HPV positive samples, respectively. The OS (overall survival) showed a not statistically significant difference in prognosis between HPV positive sand negatives (10, 47.6%, vs. 4, 44.4%; *p*-value = 0.25). *Conclusions*: HPV-DNA and p16^INK4a^ positivity confirmed the role of HPV in anal carcinoma. Our findings could support the implementation and scale-up of HPV vaccination in males and females to decrease the incidence of HPV-associated cancers. Further studies are needed to better clarify the prognostic role of HPV/p16 status.

## 1. Introduction

Anal cancer, which accounts for 4% of all lower gastrointestinal tract tumors, caused 48,541 new cases and 19,129 deaths in 2018 worldwide [1]. Its incidence and mortality trends have been steadily increasing, mainly in high-income countries [2].

Anal intercourse, multiple sexual partners [2], and early onset of sexual relationship were associated with an increased risk of acquiring Human Papillomavirus (HPV) infection, which is recognized as a cause of anal cancer (~90% of anal cancer are HPV-positive) [3]. HPV-attributed carcinogenesis is mediated by the early genes E6 and E7, which immortalize and transform host cells by inhibition of tumor suppressor activity of p53 and pRb proteins, respectively. Furthermore, E7 protein affects other cellular pathways including the over-expression of cyclin-dependent kinase inhibitor p16, which normally acts as a cell cycle regulator and tumor suppressor [4]. E6/E7 and p16 expression are considered as surrogate biomarkers in HPV-related diseases. However, the sensitivity and sensibility of these tests remains unclear and thus it is necessary to address several knowledge gaps prior their application in diagnostic tools.

In light of relevant similarities between anal and cervical cancers, numerous countries have adopted HPV-vaccinations for both sexes. It has been estimated that >95% of HPV-associated anal cancers are potentially preventable by nonavalent-HPV vaccine [5]. Although evidence of HPV vaccine in men are scanty, a recent systematic review conducted by Harder et al. showed a high effectiveness of HPV vaccination in naïve men with a reduction of 61.9% and 46.8% in anal intraepithelial lesion grade 2 and 3, respectively [6]. A potential therapeutic role of HPV vaccine, as adjuvant therapy after surgical treatment, was also suggested by several studies, which reported a reduction of recurrence in patients previously treated for anal cancer [7].

A higher incidence of anal cancer was described in men who have sex with men (MSM), people who are HIV-positive and immunocompromised (non-HIV) patients, and women with a previous HPV-related disease (i.e., cervical intraepithelial neoplasia or cancer) [8]. However, epidemiological differences can depend on the recruited population, tumor stage, and diagnostic methods.

Since the relationship between anal dysplasia and the HPV infection was proved, new classification criteria were adopted based on nomenclature used in cervical cancer (i.e., anal intraepithelial lesion (AIN) I, II, II). High/low grade squamous intraepithelial lesion (HSIL/LSIL), a precursor of anal cancer, are usually HPV-positive with a prevalence of HPV-infection, mainly HPV-16 genotype, that increases in cases of severe lesions [3,9,10]. Anal adenocarcinomas and squamous cell carcinomas (SCC), which are the histological types identified in anal cancer, can show variable HPV prevalence [11,12].

To the best of our knowledge, no previous studies have investigated the relationship between HPV infection and anal cancer in the general population in Italy. On this basis, an observational, retrospective single-center study was conducted in an Italian university hospital to describe the prevalence of HPV infection, its genotypes and positivity to biomarkers of HPV infections E6 transcript and p16 proteins in samples of anal cancer diagnosed between 2002–2018.

## 2. Methods

We conducted an observational, retrospective study on patients with a diagnosis of anal cancers at the tertiary-care university hospital in Sassari, Italy. Anal cancers are classified as anal if their epicenter is ≤2 cm from the dentate line. An ad hoc electronic form was prepared to collect demographic, clinical, and epidemiological variables in order to assess their relationship with anal cancer in patients with and without HPV infection.

### 2.1. Histopathological Evaluation

Histological sections of anal cancer samples were performed from the archived formalin-fixed paraffin-embedded (FFPE) specimens. In total, 10 consecutive sections of 3 μm were cut from 30 FFPE blocks. Pathologists evaluated hematoxylin and eosin stained sections to confirm diagnosis and tumor staging, which follows the recommendations of the classification system of the American Joint Committee on Cancer (AJCCC) [13]. Anal cancer was classified as G1 if well differentiated (low-grade), as G2 if moderately differentiated (low-grade), as G3 if poorly differentiated (high-grade), and as G4 if undifferentiated (high-grade).

### 2.2. p16 Immunohistochemistry

Immunohistochemistry was performed for p16 on formalin-fixed paraffin-embedded tissue sections using the kit CINtec p16 Histology (Ventana Medical Systems, Inc. Tucson, AZ, USA), according to manufacturer’s instructions [14]. CINtec Histology is a qualitative immunohistochemistry (IHC) test using mouse monoclonal anti-p16 antibody clone E6H4.

Cases were positive if a sequence of proliferating cells showed a strong positive signal. Although strongly positive, few isolated cells cannot confirm the positivity of a case [15].

### 2.3. HPV DNA Detection and Typing

Prior to nucleic acid purification, samples were initially deparaffinized. Paraffin was removed when adding 320 µL of deparaffinization solution (Cat. N 19093, QIAGEN, Hilden, Germany) [16]. Nucleic acid extraction was performed using a commercially available extraction kit (AllPrep DNA/RNA FFPE Kit, Qiagen, Hilden, Germany) in accordance with the manufacturer’s instructions [17,18]. DNA and RNA samples were stored at −20 °C and tested for HPV-DNA 48 h after extraction. HPV genotyping was conducted using a multiplex polymerase chain reaction (PCR) using the commercial kit Anyplex^TM^ II HPV28 Detection (Seegene, Seoul, South Korea) [19], which can detect 19 high (i.e., 16, 18, 26, 31, 33, 35, 39, 45, 51, 52, 53, 56, 58, 59, 66, 68, 69, 73, and 82) and 9 low-risk (i.e., 6, 11, 40, 42, 43, 44, 54, 61, 70) genotypes, respectively.

### 2.4. E6-transcript Detection

Samples positive for HPV-16 were tested for E6-mRNA by reverse transcriptase polymerase chain reaction (RT-PCR), using a set of primers designed by Sotlar et al. [20] for the U1-gene (forward 5′-cagagctgcaaacaactatacatgatata-3′, reverse 5′-gttaatacacctcacgtcgcagta-3′) and for the E6 transcript (forward 5′-gaagatcaagaaggatgagctaaaaa-3′, reverse 5′-tgggagaagatggcgtacag-3′).

RNA was extracted using an AllPrep DNA/RNA FFPE Kit, (Cod. 80234, Qiagen, Hilden, Germany) [16]. A cDNA synthesis was carried out using a Quantitect Reverse Transcription Kit (QIAGEN co. 205312) following the manufacturer’s recommendations [21]. Qualitative real-time PCR was performed using SYBR Green JumpStart TaqReadyMix (Cat. Num. S9194. SIGMA-ALDRICH, St Louis, MO, USA) [22]. The PCR reaction was carried out in 25 µL of final volume containing 5 μL cDNA, 2.5 μL of water, 2.5 μL each of forward and reverse primers, 12.5 μL 2× SYBR Green JumpStart ready mix, which included Taq DNA polymerase, 3.5 mM MgCl_2_, 10 mMTris-HCl, 50 mMKCl, and 0.2 mM dNTPs. The amplification was based on an initial denaturation step at 94 °C for 2 min, followed by 40 cycles of 94 °C for 15 s, and 60 °C for 1 min. Nuclease-free water and previously tested HPV16-positive liquid-based cytology samples were added in each experiment as negative and positive controls, respectively.

### 2.5. Statistical Analysis

The following variables were collected: gender, age at diagnosis, year of diagnosis, comorbidities, histopathological classification (i.e., squamous cell carcinomas (SCC) and adenocarcinomas), HPV-DNA positivity, HPV genotype, p16^INK4a^, and E6 proteins expression. Qualitative variables were summarized with absolute and relative (percentages) frequencies, whereas quantitative variables were summarized with means (standard deviations, SD), based on their parametric distribution. The Chi-squared and Fisher exact tests were computed, when appropriate, to assess differences for qualitative variables. A student t-test was computed to detect statistical differences for quantitative variables with normal distribution. The Kaplan–Meier survival analysis was performed to assess the mortality in study subgroups. A two-tailed *p*-value less than 0.05 was considered statistically significant. All statistical analyses were performed with the statistical software STATA version 15 (StatsCorp, Prosper, TX, USA).

## 3. Results

A total of 30 patients with anal cancer, diagnosed between 2002 and 2018, were retrospectively recruited; their mean (SD) age was 64.2 (12.3) years and 16 (53.3%) were males. Characteristics of the study sample are detailed in the Table 1.

Anal cancer was mainly diagnosed in persons aged ≥55 years (22/30, 73.3%); 63.3% and 36.7% were SCC and adenocarcinoma, respectively; 43.3% of the samples were moderately differentiated (G2), followed by those well differentiated (G1, 40.0%), and those poorly differentiated (G3, 16.7%). The most prevalent comorbidities were colic adenoma and bladder carcinoma.

HPV was found in 21 (70.0%) anal samples, with the HPV-16 genotype accounting for >85% (18/21) of the positive samples. Only one case showed multiple infections caused by the genotypes HPV-16, -35, and -6. One sample was positive for mono-infection caused by HPV-35 and two cases were caused by HPV-6.

Women showed a higher HPV positivity (52.4% vs. 47.6%, respectively) and HPV-16 genotype prevalence (71.4% vs. 50%, respectively) than men. Adenocarcinomas and SCCs showed an HPV-DNA positivity of 30.4% and 66.7% (*p*-value = 0.69), respectively. No statistically significant HPV differences were found according to age and year of diagnosis.

All (5, 23.8%) poorly differentiated anal samples were HPV-positive, even if the result was not statistically significant (*p*-value > 0.05).

Positivity of p-16^INK4a^ was found in ~40% of the cases, with a statistically significant difference between HPV-DNA positive and negative samples (*p*-value = 0.004). All cases positive for p16^INK4a^ were HPV-positive. The association between p16^INK4a^ and HPV-DNA positivity was found in 12 (57.1%) anal cancers. SCC and adenocarcinomas were positive for p16^INK4a^ in 36.8% (7/19) and 45.5% (5/11) of the cases, respectively. All HPV-negative samples (9, 100%) were p16^INK4a^ negative.

E6 transcripts were found in 5 (27.8%) HPV-16 positive samples. Although samples of other comorbidities were negative for high- or low-risk HPV genotypes, anal HPV-positivity was found in 7 (77.8%) patients with comorbidities.

**Figure 1:**
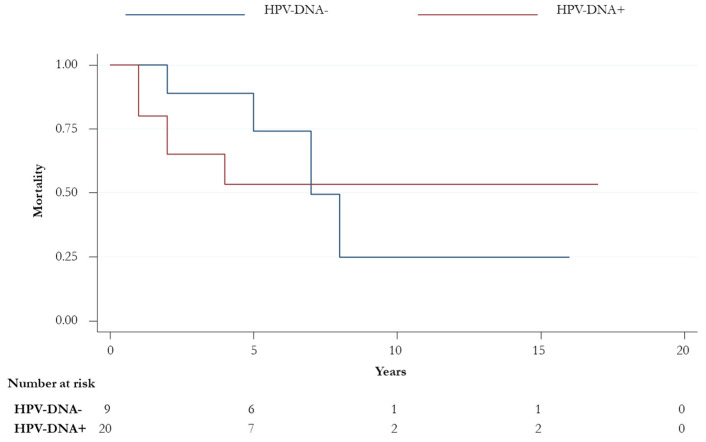
Overall survival for HPV-positive and -negative patients (*p*-value = 0.72).

**Figure 2:**
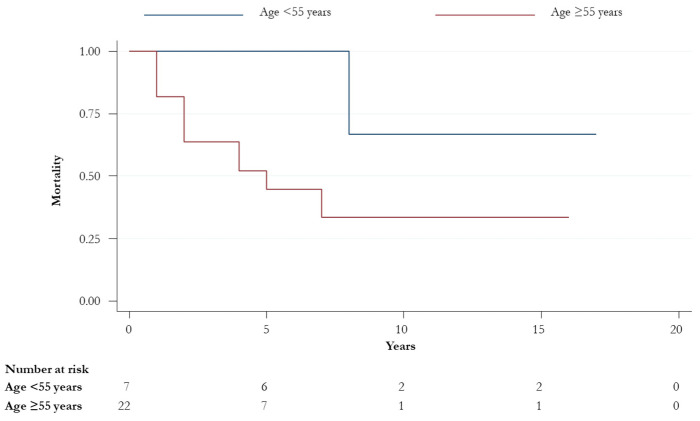
Overall survival for patients aged < and ≥55 years (*p*-value = 0.03).

A total of 16 (53.3%) patients were alive at the end of the follow-up; 56.2% (*n* = 9) were male and more than half (11, 68.8%) had a diagnosis of SCC histological subtype. Among the 14 patients who died during the follow-up, 10 (10/14, 71.4%) were HPV-positive. The overall survival (OS) did not significantly change when HPV-DNA positivity, p16 status, gender, and histological subtypes stratified patients. No statistically significant differences were observed in OS and OS at five-years between HPV-positive and -negative groups (14, 47.6%, vs. 4, 44.4%; *p*-value > 0.05) (Figure 1). However, an improved statistically significant OS was found in younger patients (75% vs. 44% in those aged <55 vs. ≥55 years, respectively; *p*-value = 0.03) (Figure 2).

## 4. Discussion

The HPV prevalence in 30 anal cancer samples, diagnosed between 2002 and 2018, in an Italian university hospital was 70.0%, with most of them associated with the HPV-16 genotype (85.7%).

Although global data estimated a higher prevalence of anal cancer in women [23], our cohort did not show any statistical difference. Furthermore, in agreement with previous reports, we found a slightly higher prevalence of SCC cases [24]. The mean age of the entire cohort is comparable to the estimates found in other studies, as well as the higher prevalence of adenocarcinoma in older patients (80% of the cases diagnosed in those aged ≥50 years) [24,25].

The overall HPV prevalence observed in our study, is slightly lower than findings of a systematic review and meta-analysis conducted by Lin et al., where prevalence of HPV infection reached 80% in all groups (i.e., stratified by gender and HIV-status), whereas it ranged from 42% to 76% from normal cytology to high-grade intraepithelial lesions [10]. Nevertheless, HPV prevalence was higher in other studies, owing to different virologic and histological methods [3,26]. Several studies suggested multiple etiologies associated with the occurrence of anal cancer, apart from HPV infection. A case-control study showed that benign lesion and prolonged inflammation were associated with an increased risk of developing anal cancer. Smoking, immunocompromised status, and sexual behavior remain the main etiological factors in anal cancer [27].

Ravenda et al. (2014) [28] found HPV positivity in 84% of anal SCC FFPE samples, whereas a study on pre-cancerous lesions (i.e., AIN2/3) diagnosed in Italian and foreign born patients showed a lower HPV prevalence (37.1%) [29].

The higher prevalence of the HPV-16 genotype was similar to the high prevalence described in other Italian studies, where it was the most prevalent genotype in anal and cervical cancers [30,31], probably for its elevated circulation and genital mucosal tropism.

The HPV-DNA prevalence was similar in both histological subtypes (i.e., SCC 73.7% and adenocarcinoma 63.6%), differing from other findings where it was proven that adenocarcinoma had a low HPV prevalence. However, the low percentage of adenocarcinoma in other studies might have underestimated the impact of HPV infections [24,26].

A higher HPV positivity was found in women (11/14, 78.5%), which can probably be explained by a persistent cervical HPV infection, which can increase the risk of anal HPV transmission [32,33]. However, missing information on cervical HPV infection of our female patients cannot confirm that hypothesis.

Another remarkable finding of this study was the positivity of p16^INK4a^ in more than half of HPV positive cases and in comparison with its negativity in HPV-negatives, showing its high specificity and strong association with the carcinogenesis mediated by the inhibition of p53 and pRb tumor suppressor proteins. Our results confirmed that p16 ^INK4a^ over expression could be adopted as an immuno-histochemical prognostic marker, as reported in head and neck HPV-associated cancers [34,35].

Probably, the poor sample size did not prove the statistically significant difference in terms of OS between the HPV-positives (Figure 1). However, as demonstrated by Razzaghi et al., who showed a decreased OS in older patients [36], we found a worse prognosis in patients aged > 55 ages. Unfortunately, disease-specific mortality was not computed and confounding variables could have influenced differences found in the OS between the two age groups.

Several study limitations, most of them attributed to the retrospective nature of the study, can be highlighted. Firstly, we did not assess a suitable sample size to prove a specific prevalence, even if all consecutive cases of anal cancer were enrolled in the study. Some known and unknown epidemiological variables (e.g., HIV status, comorbidities, sexual behavior, etc.) were not evaluated; the retrospective data collection hindered a comprehensive collection of key variables. Some statistically significant differences on the lower prevalence of HPV infection in cases of adenocarcinoma [24] and higher prevalence of HPV infection in females than males [32] were expected and not found, likely associated with the poor sample size of a mono-center study.

To the best of our knowledge, this study evaluated for the first time the role of HPV in anal cancers including demographic, histological, and molecular variables in our region. Despite the poor sample size, the etiologic role of HPV in some anal cancers can be proved by the HPV-DNA positivity and p16 over expression.

Both females and males could have a reduction of their risk after the implementation of an HPV vaccination program. However, observational prospective/retrospective and experimental studies should be carried out in the near future to better clarify the role of HPV in the carcinogenesis of anal cancer (e.g., high-risk groups) and the efficacy/effectiveness of HPV vaccines, respectively. Moreover, considering the low frequency of the disease in general population, a multicenter design could likely improve the statistical power and could discriminate statistically significant differences we did not find. The added value of point-of-care molecular methods could be showed when evaluating their diagnostic and prognostic accuracy in the routine.

## Figures and Tables

**Table 1:** Demographic, epidemiological, and clinical characteristics of the recruited patients, collected from medical records, stratified by their HPV-positivity.

Variables		*N* (%)	HPV-Positivity	HPV-Negativity	*p*-Value
Gender	Male, *n* (%)	16 (53.3)	10 (47.6)	6 (66.7)	0.44
	Female, *n* (%)	14 (46.7)	11 (52.4)	3 (33.3)	
Mean age (SD), years		64.2 (12.3)	63.7 (12.2)	65.4 (13.1)	0.72
Age ≥55 years, *n* (%)		22 (73.3)	15 (71.4)	7 (77.8)	1.00
Civil status, *n* (%) *	Married	18 (78.3)	11 (73.39	7 (87.5)	1.00
	Single	4 (17.4)	3 (20.0)	1 (12.5)	
	Divorced	1 (4.4)	1 (6.7)	0 (0.0)	
Occupational status, *n* (%) *	Unemployed	7 (35.0)	6 (46.2)	1 (14.3)	0.43
	Employed	5 (25.0)	3 (23.1)	2 (28.6)	
	Retired	8 (40.0)	4 (30.8)	4 (57.1)	
Suspected clinical diagnosis of carcinoma, *n* (%)		28 (96.6)	20 (95.2)	8 (100.0)	1.00
Suspected clinical diagnosis of adenoma, *n* (%)		1 (3.5)	1 (4.8)	0 (0.0)	1.00
Suspected clinical diagnosis of condyloma, *n* (%)		3 (10.3)	2 (9.5)	1 (12.5)	1.00
Cancer diagnosed before 2010, *n* (%)		19 (63.3)	13 (61.9)	6 (66.7)	1.00
Histopathological results, *n* (%)	Adenocarcinoma	11 (36.7)	7 (30.4)	4 (40.0)	0.69
	Squamous Cell Carcinoma	19 (63.3)	14 (66.7)	5 (55.6)	
Histopathological grading (AJCC), *n* (%)	G1	12 (40.0)	10 (47.6)	2 (22.2)	0.25
	G2	13 (43.3)	6 (28.6)	7 (77.8)	0.02
	G3	5 (16.7)	5 (23.8)	0 (0.0)	0.29
HPV-DNA positivity, *n* (%)		21 (70.0)	21 (100.0)	0 (0.0)	-
HPV-16		18 (60.0)	18 (85.7)	0 (0.0)	-
HPV-35		2 (6.7)	2 (9.5)	0 (0.0)	-
HPV-6		3 (10.0)	3 (14.3)	0 (0.0)	-
p-16 immunohistochemistry positivity, *n* (%)		12 (40.0)	12 (57.1)	0 (0.0)	0.004
E6 gene positivity, *n* (%)		5 (33.3)	5 (100.0)	0 (0.0)	-
Comorbidity, *n* (%) *	Colic adenoma	6 (66.7)	5 (71.4)	1 (71.4)	0.25
	Bladder carcinoma	2 (22.2)	2 (28.6)	0 (0.0)	
	Others	1 (11.1)	0 (0.0)	1 (50.0)	
Mortality, *n* (%)		14 (46.7)	10 (47.6)	4 (44.4)	1.00
Five-year mortality, *n* (%)		12 (40.0)	10 (47.6)	2 (22.2)	0.25

* Denominators of the collected variables can change based on the available information retrieved from the medical files.
